# Omega‐3 Fatty Acids as a Neuroprotective Agent: Behavioural and Molecular Improvements in STZ‐Induced Neurodegeneration

**DOI:** 10.1002/alz70855_100940

**Published:** 2025-12-23

**Authors:** Facundo Peralta, Ana Abril Vidal Escobedo, Juliette López Hanotte, Juan Ignacio Posada, Joaquín Pardo, Paula Cecilia Reggiani

**Affiliations:** ^1^ National Council of Scientific and Technical Research (CONICET/UNLP), La Plata, Argentina; ^2^ Facultad de Ciencias Médicas (UNLP), La Plata, Argentina

## Abstract

**Background:**

As life expectancy continues to rise, neurodegenerative disorders like AD increasingly threaten global health, impacting both patients and healthcare systems. These conditions are characterized by the loss of neurons and progressive cognitive decline, making it crucial to identify novel therapeutic approaches. Animal models, such as the streptozotocin (STZ)‐induced neurodegeneration model in rats, offer an important platform for studying potential interventions.

Omega‐3 (ω‐3) fatty acids, known for their beneficial effects on brain health, have been suggested to counteract neurodegeneration by modulating inflammatory pathways and promoting neuroplasticity. However, further research is needed to clarify their mechanisms of action and benefits. In this study, we assess the role of oral ω‐3 supplementation in alleviating behavioural and molecular changes in the hippocampus (hc), a key region affected in neurodegenerative conditions. By using the STZ rat model, we aim to provide insights into the therapeutic potential of ω‐3 in combating neurodegenerative processes.

**Method:**

On week 0, animals were randomly grouped (*n* = 8/group) into SHAM, STZ, and STZ+ω‐3, and received icv‐artificial cerebral spinal fluid (SHAM) or STZ (STZ/STZ+ω‐3) (3mg/kg) bilaterally. Among week 1 and 12, STZ+ω‐3 rats were treated daily with ω‐3 (Fish oil; 30% ω‐3 EPA and DHA; 150 µl: 44,5 mg ω‐3/rat). Two weeks before euthanizing, behavioural tests were performed. Immature neurons, microglia and astrocytes in the hc were examined using immunohistochemical labelling for DCX, Iba1 and GFAP, respectively.

**Result:**

ω‐3 treatment did not improve species‐typical behaviour (Marble burying test). However, there was a significant improvement in short‐term object recognition memory, although this effect was not observed in long‐term memory (Object recognition test). In the forced swimming test, ω‐3 treatment partially alleviated depressive‐like behaviour. At the molecular level, ω‐3 treatment did not affect immature neurons. No differences were found in microglial population, but a partial modulation of astrocytes was observed, with decreased GFAP+ cell immunoreactivity.

**Conclusion:**

These results indicate that while ω‐3 may offer therapeutic benefits in neurodegenerative conditions, its effects are selective and may not address all pathological features. Further studies are needed to fully understand its mechanisms and optimize its therapeutic potential.

